# Bing–Neel Syndrome: An Unknown GCA Mimicker

**DOI:** 10.1155/2024/2043012

**Published:** 2024-08-12

**Authors:** Arifa Javed, Sadia Arooj Javed, Barbara Ostrov, Jiang Qian, Khoa Ngo

**Affiliations:** ^1^ Albany Medical Center Department of Rheumatology, Albany, USA; ^2^ Punjab Medical College Department of Internal Medicine and Pediatrics, Faisalabad, Pakistan; ^3^ Albany Medical Center Department of Pathology, Albany, USA

## Abstract

Giant cell arteritis (GCA) is a chronic granulomatous vasculitis of medium and large arteries leading to cranial and extracranial manifestations. Temporal artery biopsy is considered the gold standard; however, its sensitivity is low at 47%. We report a unique case of Bing–Neel Syndrome (BNS) presenting as biopsy-proven GCA. BNS is a rare complication (1%) of Waldenstrom Macroglobulinemia (WM), which results from infiltration of lymph plasmacytoid cells and plasma cells into the central nervous system. A 77-year-old female with a past medical history of glaucoma, hypertension, diabetes, and chronic ocular ischemic syndrome in her right eye presented with progressive left eye vision loss for 5 days. Fundoscopic examination was notable for pseudophakic pseudopallor but no optic disc edema. Intraocular pressure was >40 and normalized after acetazolamide. The patient was started on pulse dose steroids by her neuro-ophthalmologist. She was discharged home on 60 mg of prednisone. At follow up with her neuro-ophthalmologist, new dot blot hemorrhages in the left eye were noted and she was readmitted for pulse dose of intravenous methylprednisolone. Temporal artery biopsy was consistent with GCA spectrum. Work up revealed paraproteinemia and subsequent bone marrow biopsy demonstrated WM. The patient was treated for her WM and her ophthalmic complications stabilized.

## 1. Introduction

Giant cell arteritis (GCA) is the most common systemic autoimmune-mediated vasculitis in adults over the age of 50. It is a granulomatous vasculitis of medium and large arteries leading to cranial and extracranial manifestations. It can involve extracranial branches of carotid artery, subclavian, axillary, brachial, aorta, and less commonly iliac and femoral arteries. Temporal artery biopsy remains the gold standard for diagnosis, although its sensitivity is reported to be only 47% [1]. It is part of the ACR criteria for diagnosis of GCA along with the “halo sign” on ultrasonography [2]. Therefore, mimickers of GCA need to be ruled out prior to making a definitive diagnosis Bing–Neel Syndrome (BNS) is a rare neurological complication of Waldenström's Macroglobulinemia (WM), characterized by the infiltration of lymphoplasmacytic cells into the central nervous system (CNS), affecting approximately 1% of WM patients [3]. This current report highlights a unique case where BNS presented as biopsy proven GCA, challenging the conventional diagnostic and therapeutic pathway and emphasizing the importance of understanding expected characteristic histopathology in such cases. Patient consent was obtained to publish this report.

## 2. Case Presentation

**Figure 1:**
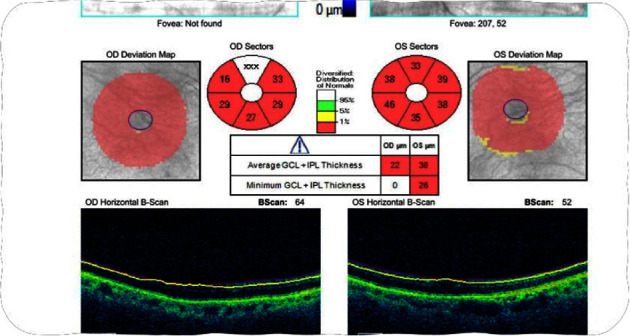
OCT of Ganglion layer.

**Figure 2:**
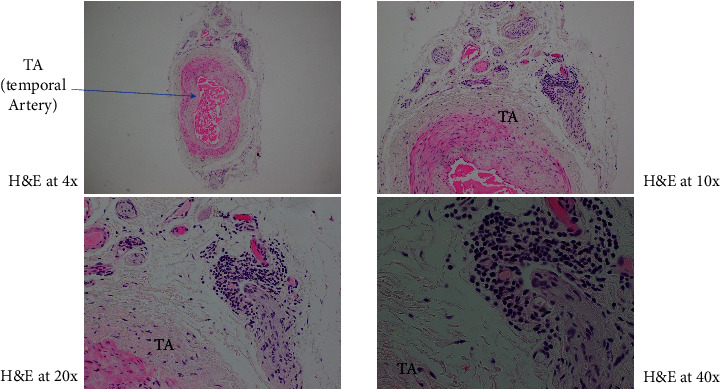
Microfocus of mononuclear cells in the adventitia around vasa vasorum, composed mainly of small-sized lymphocytes.

A 77-year-old female with a history of glaucoma, hypertension, diabetes, and chronic ocular ischemic syndrome presented with a 5-day history of progressive vision loss in her left eye. Initial fundoscopic examination revealed “pseudophakic pseudopallor” without optic disc edema. OCT (ocular coherence testing) was remarkable for reduced RNFL (retinal nerve fibre layer) as seen in Figure 1. Intraocular pressure exceeding 40 mmHg, which normalized following administration of acetazolamide. Patient was referred to the Emergency Department by her neuro-ophthalmologist. Patient was started on methylprednisolone 1 g daily for 5 days followed by 60 mg of prednisone. Patient was discharged on this dose. On follow up with neuro-ophthalmology, new dot blot hemorrhages were noted in left eye. Patient was readmitted for pulse dose steroids for 3 days. Her initial labs were remarkable for CRP 23.7 mg/L (<8), ESR 62 mm/hr (0–29). Work up for GCA mimickers revealed quantitative IgM 4000 (52–257), serum immunofixation with IgG and IgM kappa, serum FLC+ with elevated free kappa 34, elevated lambda 4.5, and elevated ratio 7.58 (<1.68). Temporal artery biopsy performed on previous admission showed changes consistent with GCA including mural lymphohistiocytic inflammation, mostly adventitia based, with focal involvement of the outer muscle layer and its elastic lamina, while largely sparing the intima, media, and internal elastic lamina, but without giant cells (Figure 2).

**Figure 3:**
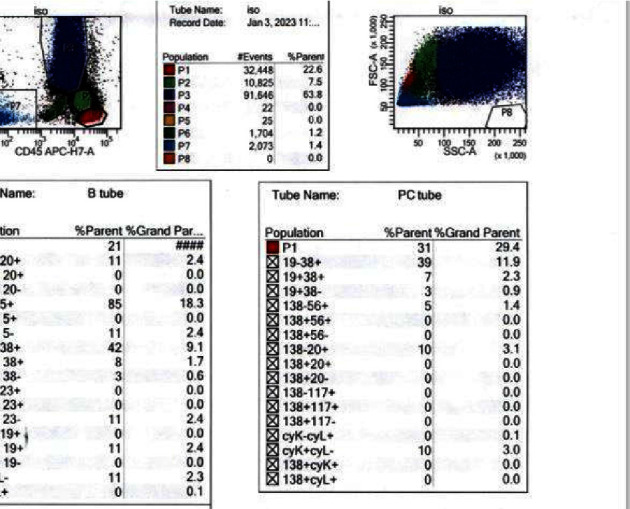
Flow cytometry. Temporal artery (TA), hematoxylin and eosin (H & E), ocular coherence test (OCT), P1-lymphocytes, P2-monocytes, P3-granulocytes, P7-plasma cells.

**Figure 4:**
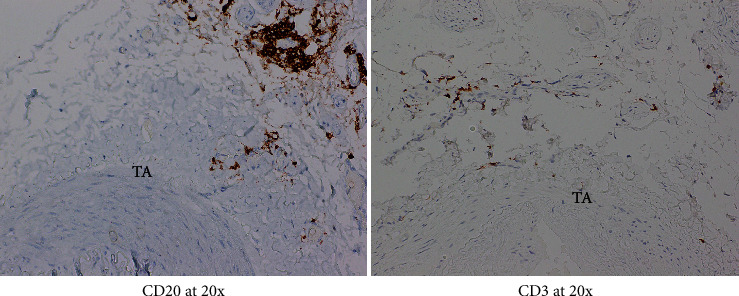
Microfocus showing most lymphocytes are CD20-positive B cells, with rare CD3-positive T cells.

**Figure 5:**
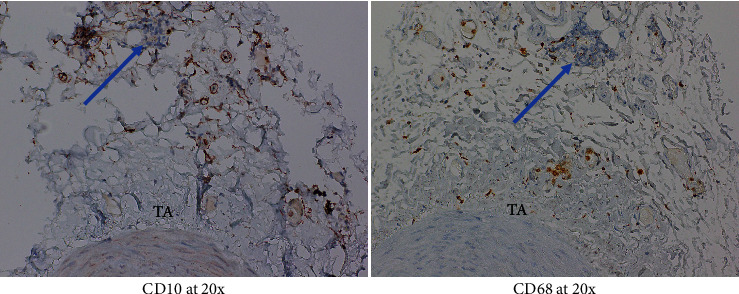
Lymphocytes in this microfocus are negative for CD10 and CD68. There is small numbers of histiocytes (CD68+) in adventitia.

Given patient's plasma cell dyscrasia and to work up the differentials for GCA, bone marrow biopsy was requested. It confirmed the diagnosis of WM, with flow cytometry identifying 2% aberrant B cells with a phenotype suggestive of lymphoplasmacytic lymphoma (Figure 3). After discussion with pathology, the temporal artery biopsy was revisited, and additional stains performed. These demonstrated a mononuclear cell infiltrate composed of small numbers of scattered small-sized lymphoid cells (positive for CD20 and kappa light chain, Figure 4) and rare histiocytes (positive for CD68, Figure 5) without forming a solid mass lesion. The changes were consistent with small B cell lymphoma involving temporal artery, causing clinical temporal arteritis pattern of injury. Patient was treated with 6 cycles of bendamustine and rituximab which halted the ophthalmic symptoms from worsening.

## 3. Discussion

The diagnosis of GCA is challenging due to its variable presentation and the limitations of temporal artery biopsy. GCA is characterized by transmural inflammation, intimal hyperplasia, and fragmentation of elastic lamina [4]. Granulomatous inflammation consisting of macrophages and CD4 cells is seen in the vessel wall. Even though giant cells have been historically considered pathognomonic for GCA, they are only seen in half of the biopsy results [4]. In GCA, CD4 cells play the main role in pathogenesis, which are normally not present in healthy arteries (refer to Table 1).

It is postulated that they permeate adventitia through vasa vasorum [5]. CD4 cells are polarised towards Th1 and Th17 cells in the milieu rather than Treg and Th2 [5, 6]. They produce interferon gamma and IL17, respectively, which lead to cytokine production and subsequent neo—angiogenesis and inflammation of arterial wall leading to narrowing of vascular lumen. Unlike the granulomatous inflammation seen in GCA, BNS is characterized by the infiltration of LPCs and plasma cells within the CNS, including the brain, spinal cord, and lepto meninges [7].

The immunophenotype characteristic of BNS includes positive B cell surface markers (CD19, CD20, CD79a, and CD79b), positive plasma cell markers (CD138 and CD38), and variable CD27, CD52, CD5, and CD3 expression [7]. Kappa light chain restriction is usually observed though lambda light chain restriction has been occasionally reported. Hence, immunohistochemical staining of tissues play a crucial role in helping to diagnose direct infiltration by the LPC cells.

Manifestations of BNS include gait deficits and balance abnormalities in 48% of the patients [8]. It can also present with sensory and motor deficits, headache, altered mental status, and cranial nerve abnormalities [2]. Cases of BNS have been reported demonstrating vision changes through involvement of cranial nerves V, VII, VIII, optic nerve, and optic chiasma [9], but none showing vision loss through infiltration of temporal artery. Our case is the rare example of such complex manifestations, which is, to the best of our knowledge, the first report of BNS mimicking GCA. BNS is typically diagnosed after identifying WM, but in our case, it was the presenting feature which makes this case even more unique [10]. It is important to consider BNS along with other complications associated with WM, such as hyperviscosity syndrome and neuropathy related to antimyelin-associated glycoprotein antibodies, hence patients present with atypical symptoms [7].

GCA presents with myriad of nonspecific symptoms including headache, jaw claudication, weight loss, fatigue, fever, and vison loss. Many symptoms overlap in patients with lymphoma or clonal haematological disorder. Therefore, a high clinical suspicion is required in such cases. In our case, at initial presentation of GCA symptoms, the patient relapsed quickly while being treated with high-dose prednisone which alerted us to consider an alternative diagnosis that was mimicking GCA. Identifying the correct diagnosis in our patient led to directed therapy and was crucial to maximize the patient's outcome.

## 4. Conclusion

This case illustrates the diagnostic challenge posed by BNS when it presents with clinical features mimicking GCA. It underscores the importance of considering a broad differential diagnosis in patients with atypical presentations and highlights the role of immunohistopathological evaluation. Clinicians should adopt an interdisciplinary approach to diagnose and manage such cases as it can influence treatment considerations and prognosis.

## Figures and Tables

**Table 1:** Comparison of characteristics and findings of our patient with typical GCA patient.

	Patient with BNS mimicking GCA	Typical GCA patients
Clinical presentation	Vision loss, retinal hemorrhages, ESR 62 SPEP IgM total 4000	Visual field cut Sudden vision loss vs amaurosis fugax
Histological findings	Mural lymphohistiocytic inflammation in adventitia with focal involvement of the outer muscle layer and elastic lamina, sparing the intima, media and internal elastic lamina, but without giant cells	Giant cells in 50%
Immunohistological findings	Mononuclear cell infiltrate with scattered small-sized CD20 and kappa light chain + lymphoid cells and rare CD68 + histiocytes, without forming a solid mass consistent with small B cell lymphoma	Predominant CD4 cells and macrophages
Constitutional symptoms	Can be present	Present
Response to GCA therapy	Poor initial response with progressive vision changes	Good to excellent response to high dose glucocorticoids and/or biologic agent

## Data Availability

No data were used to support this study.
